# Virtual Screening–Derived Computational Lead Candidates for MAO-B Inhibition in Parkinson’s Disease: Pathway-Informed Docking, Molecular Dynamics, DFT and ADMET Evaluation

**DOI:** 10.3390/ijms27219781

**Published:** 2026-10-30

**Authors:** Ibrahim Khater, Seifeldin Elabed, Modather F. Hussein, Khaled M. Alam-Eldein, Ahmed A. Aly, Somaia G. Hamada, Ahmed T. Elhendawy

**Affiliations:** 1 Department of Biophysics, Faculty of Science, Cairo University, Giza 12613, Egypt; ikhater@sci.cu.edu.eg; 2 Medical Biophysics Division, Physics Department, Faculty of Science, Helwan University, Cairo 11829, Egypt; seifeldin25@science.helwan.edu.eg; 3 Biotechnology and Genetic Engineering Department, Faculty of Science, Helwan National University, Cairo 11829, Egypt; 4 Department of Chemistry, College of Science, Jouf University, Sakaka 72388, Al-Jouf, Saudi Arabia; mfhussin@ju.edu.sa; 5 Molecular Biology and Biotechnology Department, School of Biotechnology, Badr University in Cairo, Badr City 11829, Egypt; 6 Unit of Scientific Research, Applied College, Qassim University, Buraydah 51452, Al-Qassim, Saudi Arabia; 7 Chemistry Department, Faculty of Science, Zagazig University, Zagazig 44519, Egypt; somayagameel8@gmail.com; 8 Faculty of Engineering Technology, Elsewedy University of Technology–Polytechnic Egypt, Cairo 7060010, Egypt; ahmed.taher@sut.edu.eg

**Keywords:** Parkinson’s disease, monoamine oxidase B, STRING, KEGG, virtual screening, molecular docking, molecular dynamics, density functional theory, ADMET

## Abstract

Monoamine oxidase B (MAO-B) is a clinically validated target in Parkinson’s disease (PD), but docking rank alone is insufficient to establish a CNS-directed inhibitor. We integrated MAOB-related pathway context, domain-resolved structural annotation, GNINA virtual screening, predicted blood–brain barrier (BBB) permeation, molecular dynamics (MD), density functional theory (DFT) and ADMET assessment to nominate hypothesis-stage computational candidates. A pharmacophore-derived input collection 2870 entries was subjected to virtual screening; a curated BBB-positive, favorable Vina/CNN-affinity shortlist yielded PubChem CIDs 143667968, 42876521 and 1232631. The three candidates had similar reported GNINA scores. In single 100 ns MD trajectories, CID 42876521 displayed frequent protein–ligand hydrogen bonds, whereas CID 143667968 displayed almost none and reduced aromatic-cage fluctuation. CID 143667968 also had the smallest gas-phase HOMO–LUMO gap. All three compounds carried predicted mutagenicity, carcinogenicity and hERG alerts. CID 42876521 is therefore prioritized for validation, CID 143667968 is retained as a complementary hypothesis and CID 1232631 as a backup scaffold. These results define a preliminary, multi-parameter prioritization framework.

## 1. Introduction

Parkinson’s disease (PD) is a progressive, clinically heterogeneous neurodegenerative disorder defined by bradykinesia in combination with rigidity and/or rest tremor, accompanied by a broad spectrum of non-motor manifestations. Its neuropathology includes the degeneration of nigrostriatal dopaminergic neurons and alpha-synuclein-rich Lewy pathology, but contemporary models emphasize that mitochondrial dysfunction, proteostasis failure, lysosomal biology, neuroinflammation, altered synaptic homeostasis and genetic susceptibility interact across disease stages rather than forming a single linear cascade [1,2,3,4]. Current therapy remains predominantly symptomatic. Levodopa is the most effective dopaminergic treatment, yet chronic treatment is frequently complicated by wearing-off and dyskinesia, and currently available interventions do not reliably halt neurodegeneration [5,6]. This therapeutic gap sustains interest in CNS-active small molecules that can modulate established dopaminergic pathways while retaining tractable medicinal-chemistry properties.

Monoamine oxidases A and B are flavin adenine dinucleotide (FAD)-dependent enzymes localized to the outer mitochondrial membrane. MAO-B is clinically actionable in PD because inhibition reduces the oxidative deamination of monoamines and can prolong dopaminergic signaling; selegiline, rasagiline and safinamide exemplify the pharmacological utility of this target [7,8,9,10]. A 2024 network meta-analysis of 30 trials found significant improvement in motor outcomes for several MAO-B inhibitor regimens relative to placebo, reinforcing the continuing clinical relevance of the target [11]. Brain MAO-B activity increases with age [12], and the enzyme’s role extends beyond dopamine degradation: MAO-A and MAO-B make context-dependent contributions to striatal dopamine turnover and astrocytic GABA synthesis [13], and reactive-astrocyte MAO-B, hydrogen-peroxide generation and GABA production have emerged as additional hypotheses relevant to parkinsonian neurobiology [14]. These observations strengthen the rationale for MAO-B modulation.

The target is also structurally well suited to mechanism-oriented computational analysis. Human MAO-B contains covalent FAD and a bipartite entrance/substrate cavity whose geometry controls access to the catalytic flavin [15,16,17,18]. Tyr398 and Tyr435 form an aromatic cage adjacent to the FAD isoalloxazine ring, and mutagenesis has demonstrated functional importance for this environment [19]. Ile199 and Tyr326 participate in opening and closing the junction between the entrance and substrate cavities, thereby influencing substrate and inhibitor recognition [16]. PDB 6FW0 is particularly informative because it is a 1.60 Å human MAO-B structure with covalent FAD and a bound chlorophenyl-chromone-carboxamide inhibitor (E92), providing a high-resolution reference for reversible inhibitor recognition [20]; chromones are a privileged scaffold for MAO-B inhibition [21,22]. The protein is a membrane-associated homodimer; the C-terminal region therefore has a different structural role from the catalytic core, a distinction that is relevant to soluble MD simulations [20,23].

Virtual screening couples docking and scoring with downstream filters to reduce large chemical libraries to a small number of testable hypotheses [24,25]. Docking scores, however, correlate imperfectly with measured affinity [26], so the credible nomination of a computational lead benefits from orthogonal evidence on pose persistence, protein response, electronic character and developability. Structure-based MAO-B discovery is well established, and recent studies combine docking, ADMET prediction, 100 ns MD, DFT, MM/GBSA, pharmacophore and QSAR models, MAO-A selectivity screening and simulations extending to 200–500 ns [27,28,29]. For example, a 2024 acefylline study used redocking, MD, DFT, ADMET and MM/GBSA [28], while a 2025 MAO-B selectivity workflow integrated pharmacophore modeling, QSAR, multi-database screening, 200 ns MD and MM/PBSA [29].

**Scheme 1:**
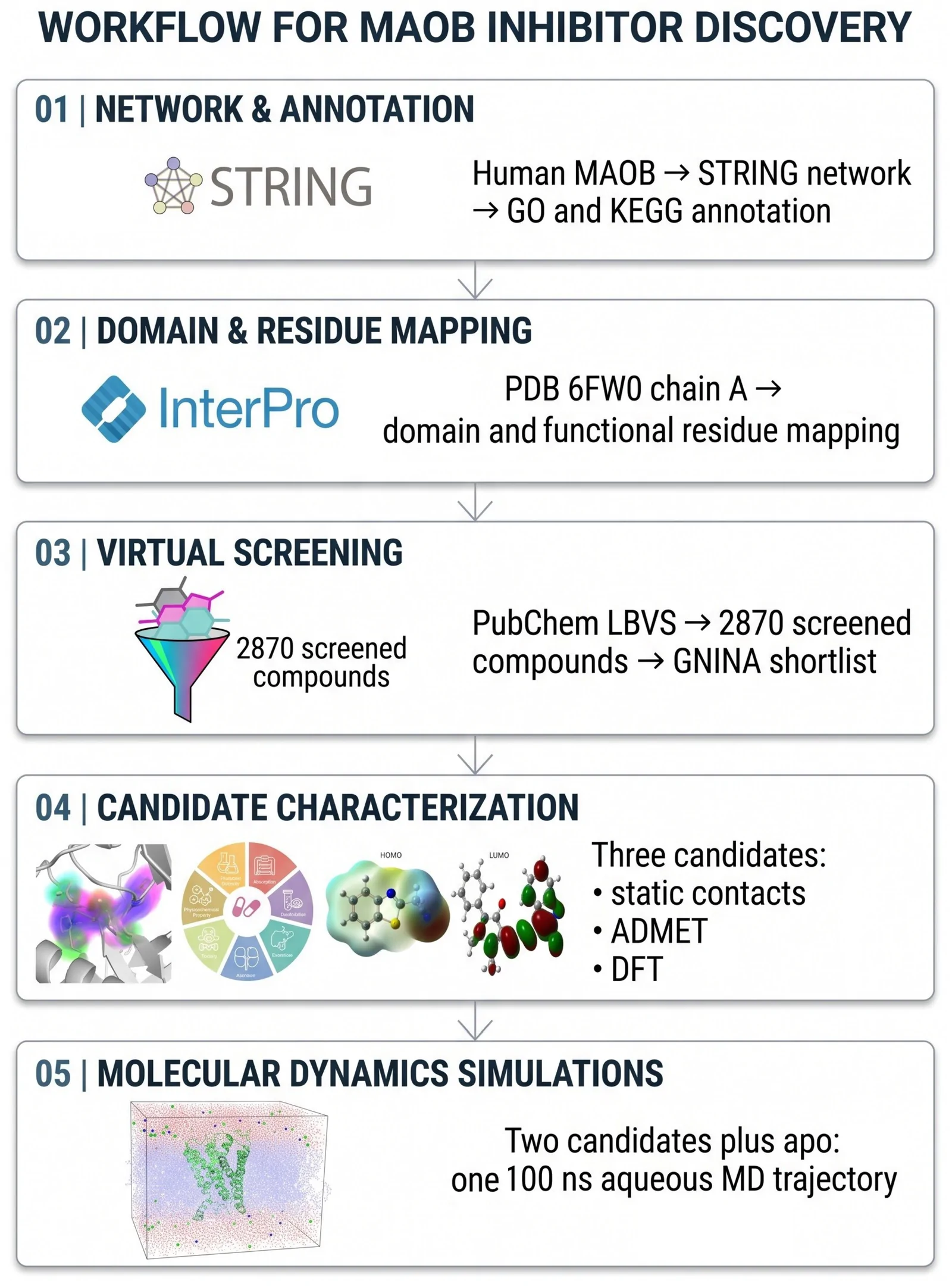
Schematic representation of the computational workflow adopted in the present study, illustrating the sequential steps of molecular docking, geometry optimization, density functional theory (DFT) calculations, molecular dynamics simulations, and ADMET prediction.

Accordingly, the present study followed a workflow proceeding from pathway context through domain annotation to docking and molecular dynamics. Before docking, a human MAOB-centered STRING network was evaluated with GO and KEGG enrichment to establish the biochemical pathway context of the target. Independent KEGG annotation distinguished curated Parkinson disease pathway membership from enrichment obtained from the MAOB-centered network [30,31]. Next, InterProScan, Pfam, PRINTS, CATH-Gene3D, SUPERFAMILY and Phobius annotations were mapped directly to 6FW0 numbering to separate the amine-oxidase/FAD catalytic architecture from the C-terminal membrane anchor [32,33,34]. Three docking-prioritized compounds were then characterized by ADMET prediction and DFT, two of them by 100 ns MD, and the combined evidence was used to propose, as hypotheses, a leading computational candidate, a complementary candidate and a back-up scaffold. The aim of this study was to nominate hypothesis-stage computational lead candidates for MAO-B inhibition by integrating pathway context, domain-resolved structural annotation, docking, MD, DFT and ADMET prediction. The overall workflow is summarized in Scheme 1.

## 2. Results

### 2.1. The MAOB-Centered Network

**Figure 1:**
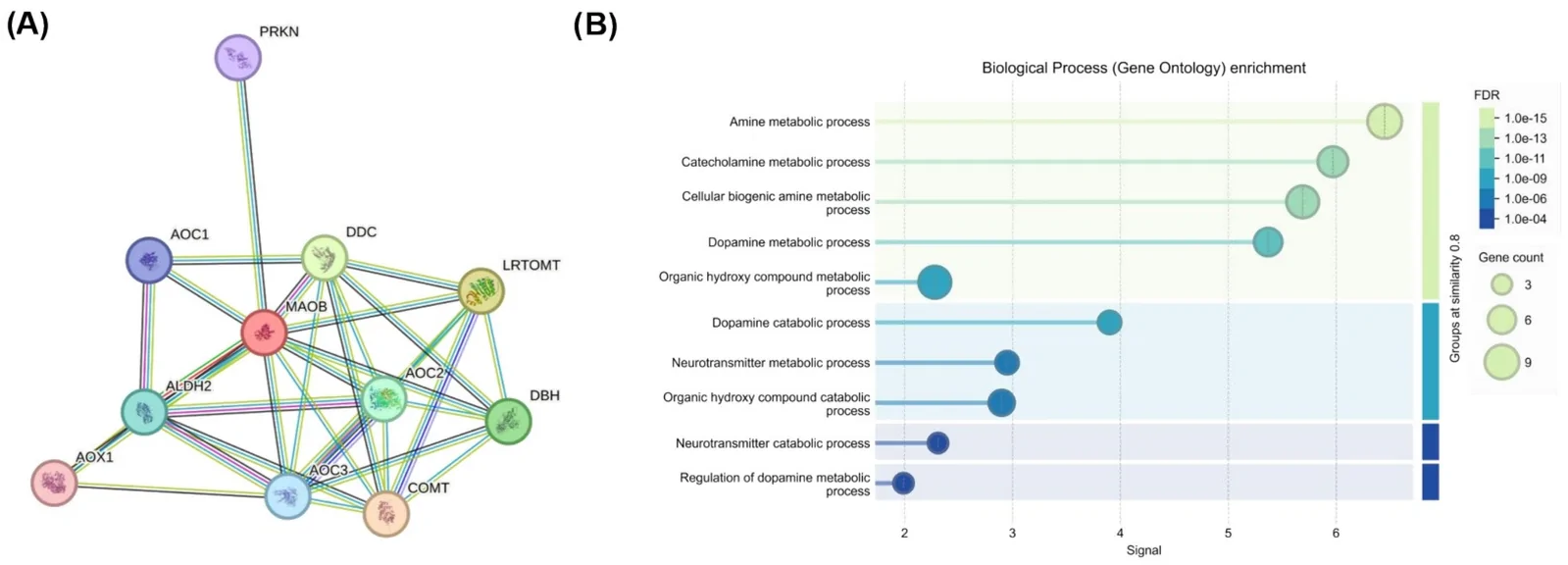
Systems-level context of human MAO-B. (**A**) STRING v12.0 functional-association network centered on MAOB and ten first-shell human proteins. Edges indicate STRING functional associations rather than necessarily direct physical binding. (**B**) Selected GO Biological Process enrichments. Bubble size represents gene count, the x-axis is the STRING enrichment signal, and the color scale represents FDR. The dominant signal converges on amine, catecholamine, dopamine, and neurotransmitter metabolism.

The MAOB-centered STRING network comprised 11 proteins connected by 32 functional associations (Figure 1A). Because MAOB was the predefined seed, it had the highest degree (10); among the remaining nodes the largest degrees were observed for AOC3 (8), AOC2, COMT and DDC (7 each), and ALDH2, DBH and LRTOMT (6 each). The strongest associations of MAOB were with COMT (0.996), LRTOMT (0.990), DDC (0.987), DBH (0.984), AOC2 (0.979) and ALDH2 (0.975).

Gene Ontology enrichment was dominated by amine-related terms: 9 of 11 proteins (81.8%) mapped to amine metabolic process, and progressively narrower terms covering catecholamine and dopamine metabolism remained highly significant (Table 1; Figure 1B). Four proteins (36.4%) mapped to the dopamine catabolic process (FDR 6.52 × 10^−8^). The simultaneous enrichment of broad and narrow terms indicates that the first-shell network is concentrated in amine- and dopamine-related metabolic functions.

**Figure 2:**
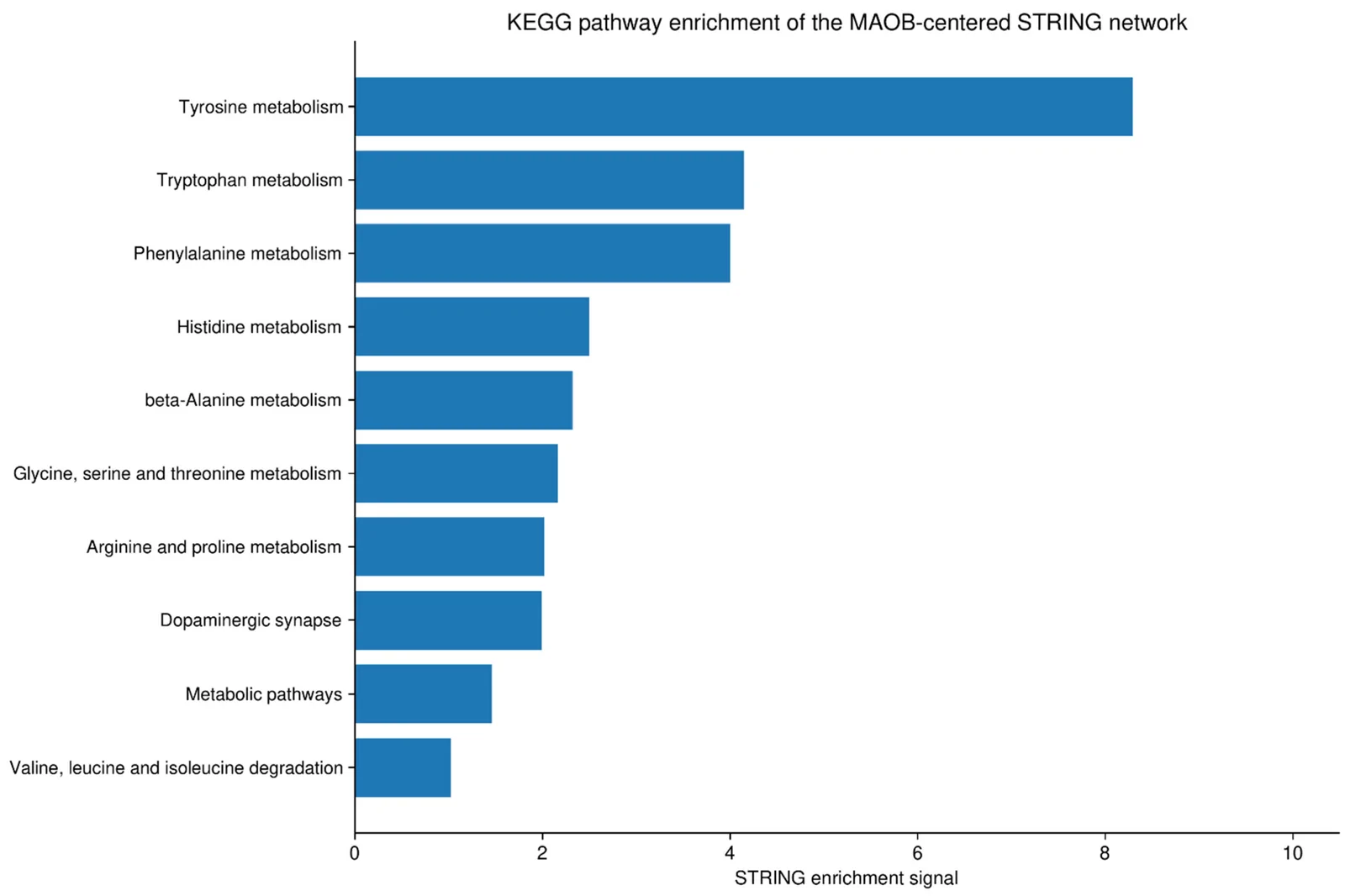
KEGG enrichment of the MAOB-centered STRING neighborhood. Bars represent the pathway-enrichment signal, while labels identify mapped network gene counts and false-discovery rates (FDRs).

KEGG enrichment provided complementary pathway context (Table 2; Figure 2). Tyrosine metabolism showed the strongest signal (8 of 11 genes; signal 8.29; FDR 1.42 × 10^−17^), followed by tryptophan metabolism, phenylalanine metabolism and the dopaminergic synapse. The broad term ‘metabolic pathways’ contained 10 of 11 proteins but had a markedly lower signal (1.46), showing that enrichment strength is not determined by gene count alone. Independently of this network-derived enrichment, KEGG maps human MAOB (hsa:4129) to tyrosine metabolism, dopaminergic synapse and Parkinson disease (hsa05012).

### 2.2. Domain Annotation

**Figure 3:**
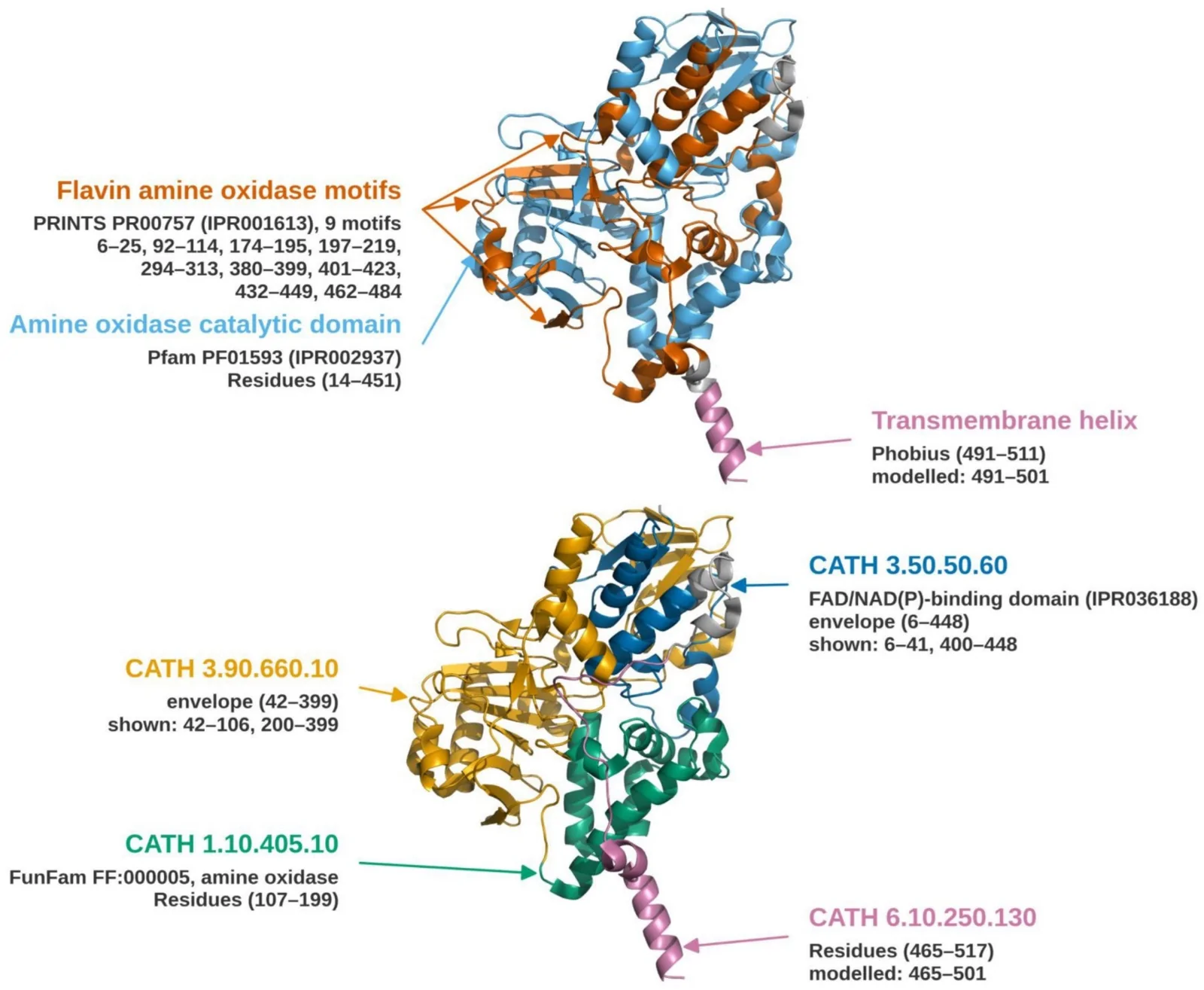
Functional and structural domain organization of human MAO-B (6FW0). The upper representation maps the Pfam amine-oxidase catalytic domain, nine PRINTS flavin-amine-oxidase motifs, and the predicted C-terminal transmembrane helix. The lower representation maps nested CATH-Gene3D assignments, including the FAD/NAD(P)-binding envelope, amine-oxidase FunFam, and C-terminal domain.

Sequence–structure annotation provided a coherent view of the full-length chain (Figure 3; Table 3). The Pfam amine-oxidase domain (PF01593) spans residues 14–451, i.e., 438 residues or 87.6% of the 500 residues modeled in chain A, and nine PRINTS flavin-amine-oxidase motifs collectively cover 192 modeled residues (38.4%). The 380–399 motif contains Cys397, which the 6FW0 LINK records connect covalently to FAD, and Tyr398, one component of the aromatic cage; the 432–449 motif contains Tyr435, the second. The two short motif windows used later for domain-resolved RMSF therefore overlap the FAD-facing recognition environment directly and do not represent arbitrary residue bins.

CATH and SUPERFAMILY produced concordant higher-order assignments. CATH 3.50.50.60 spans residues 6–448 as an FAD/NAD(P)-binding envelope; nested within it are CATH 3.90.660.10 (42–399) and the amine-oxidase FunFam 1.10.405.10 (107–199). SUPERFAMILY classified residues 1–454 within an FAD/NAD(P)-binding fold, with residues 290–400 assigned to an FAD-linked reductase C-terminal superfamily. In contrast, the extreme C-terminus lies outside the main catalytic annotation: CATH 6.10.250.130 covers 465–517, while Phobius predicts a transmembrane helix at 491–511. Only 491–501 of that helix are modeled in chain A. This distinction was important for interpreting the terminal RMSF peak in the water-solvated MD trajectories.

### 2.3. GNINA Screening

GNINA-based analysis of the reported 2870-entry pharmacophore input set [35] yielded a pragmatic shortlist of PubChem CIDs 143667968, 42876521 and 1232631 (Table 4). Selection was not based on the lowest Vina score alone: it combined favorable (more negative) Vina scores, higher GNINA CNN-affinity estimates and a positive predicted BBB-permeation classification, the latter being important for a CNS-directed Parkinson’s disease hypothesis. The selected compounds have reported Vina scores of −13.48, −13.26 and −13.15 kcal mol^−1^ and CNN-affinity estimates of 7.39, 7.08 and 7.07 pK units, respectively. Ligand efficiency (−Vina score/heavy-atom count) was calculated as a supporting size-normalized descriptor (0.50, 0.51 and 0.49 kcal mol^−1^ per heavy atom, respectively), rather than a substitute for predicted permeability or experimental binding [26,36,37,38,39].

Selection rationale and chemical classes. The initial collection was obtained by pharmacophore matching. The three retained structures are structurally distinct, halogen-containing nitrogen-heteroaromatic compounds. The supplied screening export contains a separately curated comparison with ten BBB annotations, including six marked “yes”. Among those six annotated BBB-positive entries, the three selected CIDs have the three highest tabulated CNN-affinity estimates (7.390, 7.088 and 7.072), while also retaining favorable Vina values (−13.482, −13.269 and −13.151 kcal mol^−1^).

The next three BBB-positive entries in the curated comparison were CID 46206669 (CNN affinity 6.905; Vina −13.153 kcal mol^−1^), CID 1548668 (6.826; −13.321 kcal mol^−1^) and CID 53044383 (6.717; −13.341 kcal mol^−1^). Other entries with very favorable docking scores were annotated as BBB-negative in the curated comparison (e.g., CID 71696642, Vina −15.961 kcal mol^−1^; CID 19451687, CNN affinity 7.544 pK).

### 2.4. ADMET Profiles

**Figure 4:**
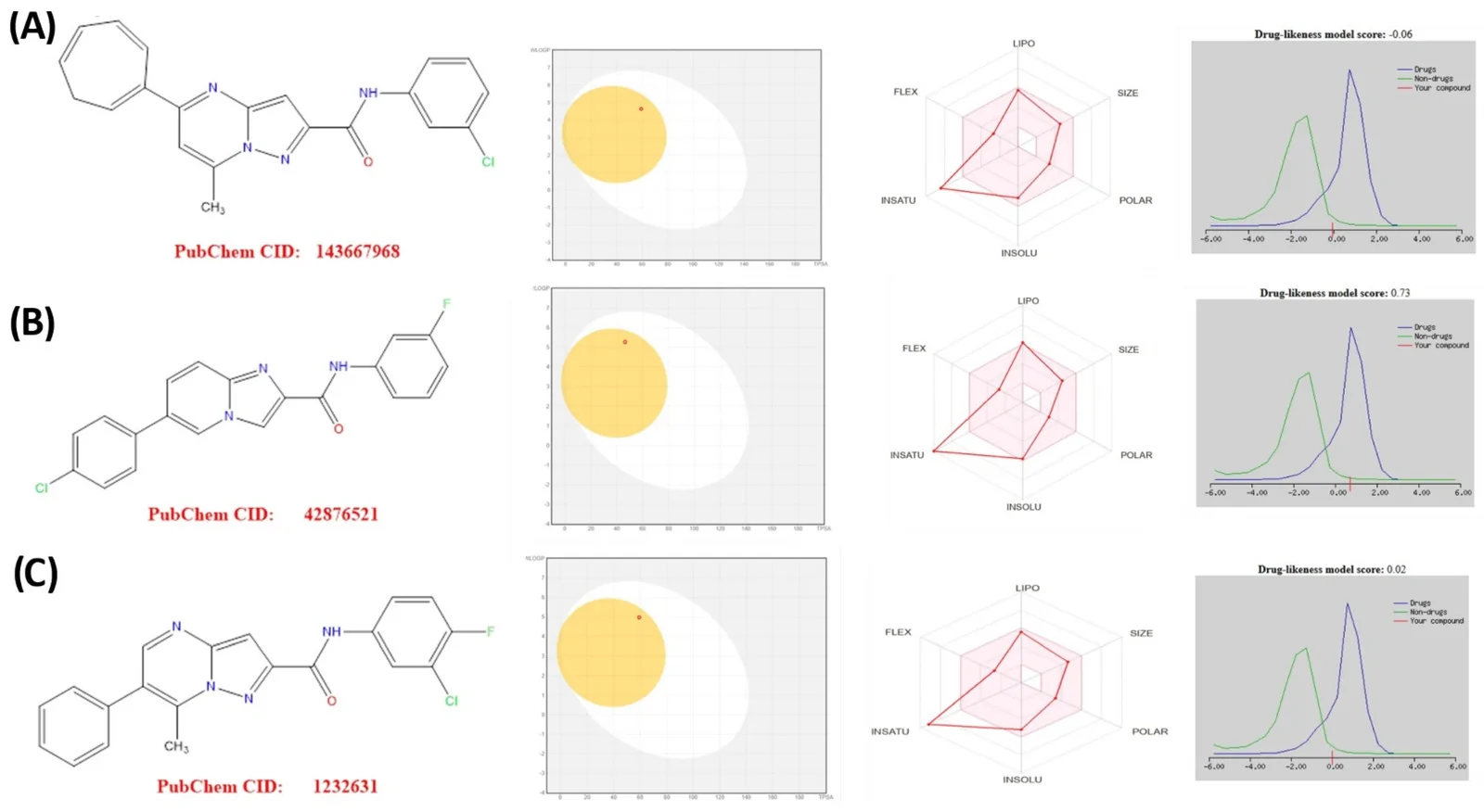
Comparative structures and in silico developability profiles of the three prioritized ligands. Rows correspond to PubChem (**A**) CIDs 143667968, (**B**) 42876521, and (**C**) 1232631, respectively. The panels combine chemical structure, BOILED-Egg distribution, SwissADME bioavailability radar, and drug-likeness score. In the BOILED-Egg plots, the yellow region (yolk) denotes the predicted BBB-permeation zone and the white region the predicted gastrointestinal-absorption zone; red dots mark the compounds. In the bioavailability radars, the pink area is the optimal range and the red line is the compound profile. In the drug-likeness plots, the blue curve is the drug distribution, the green curve the non-drug distribution and the red line the compound score.

All three prioritized compounds were classified by SwissADME as having high predicted gastrointestinal absorption and potential BBB permeability (Table 5; Figure 4). The compounds also have molecular weights below 450 g mol^−1^ and TPSA values below 90 Å^2^, which are CNS-oriented descriptive ranges [40,41], but the BOILED-Egg prediction uses its own WLOGP/TPSA decision regions [42,43] rather than these two simple values as a universal acceptance rule. The predictions differed across solubility models: ESOL values were moderately low for all three, whereas SILICOS-IT estimated particularly poor solubility for CIDs 42876521 and 1232631. CID 42876521 had the highest reported drug-likeness model score, but Ames and rat-carcinogenicity alerts and medium hERG-risk categories were predicted for all three; CID 42876521 additionally showed a positive mouse carcinogenicity prediction. Accordingly, BBB suitability and favorable docking were used for screening prioritization, while toxicity and solubility were retained as important unresolved liabilities [44].

### 2.5. Docking Poses Localize the Prioritized Ligands to the FAD-Facing Cavity with Distinct Contact Patterns

**Figure 5:**
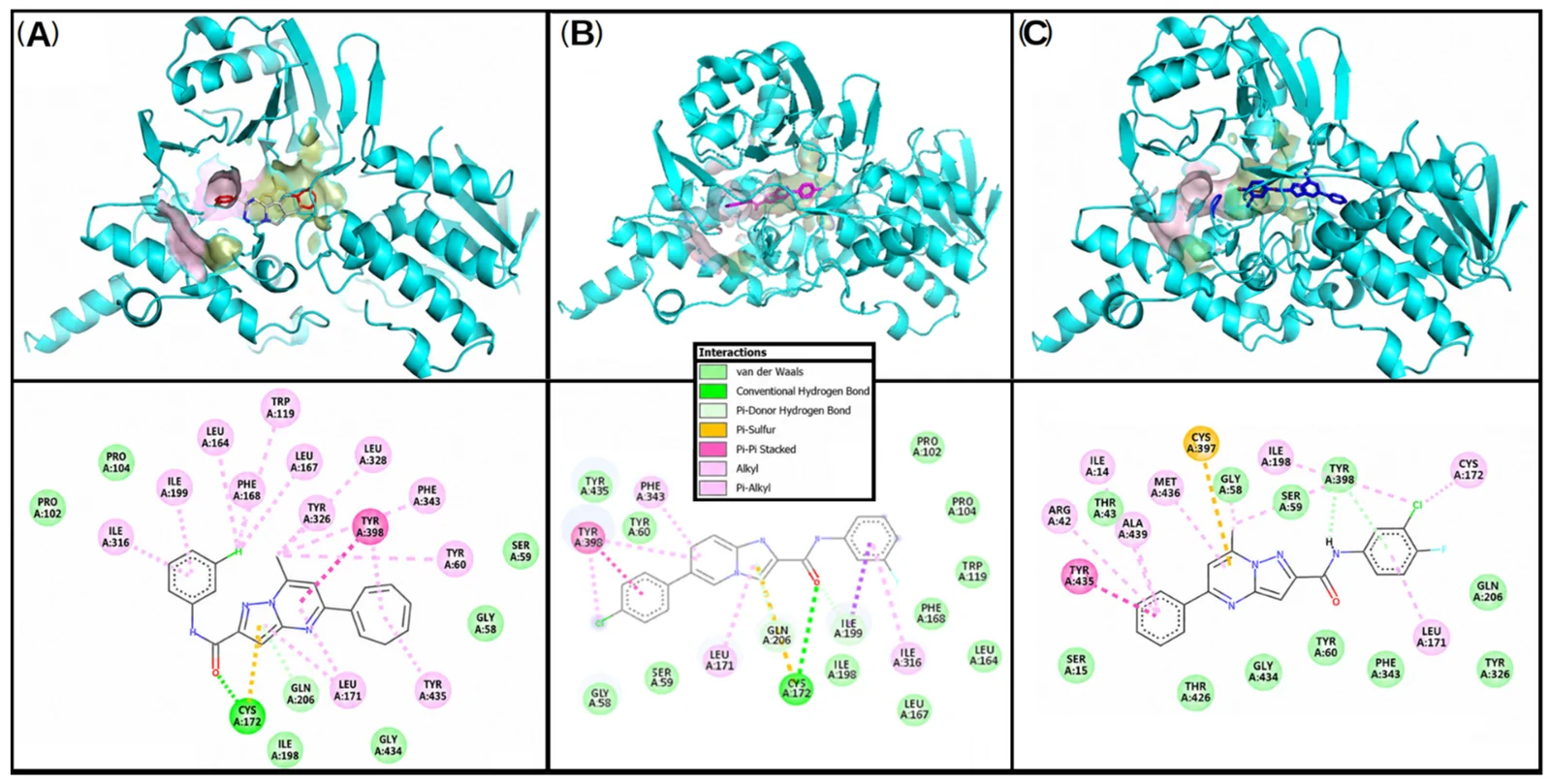
Docked binding modes and two-dimensional interaction maps of the prioritized MAO-B ligands. Upper row: three-dimensional poses of CIDs (**A**) 143667968, (**B**) 42876521 and (**C**) 1232631 in the FAD-facing cavity of MAO-B (cartoon, chain A; protein in cyan, ligand as sticks). Lower row: corresponding two-dimensional interaction diagrams in which circles denote contacting residues and are colored by interaction type (van der Waals, conventional hydrogen bond, π-donor hydrogen bond, π–sulfur, π–π stacked, alkyl and π–alkyl; see legend). Particular mechanistic attention is given to Cys172, Ile198/Ile199, Tyr326, Phe343, Cys397, Tyr398 and Tyr435.

The three-dimensional and two-dimensional interaction maps (Figure 5) position the shortlisted molecules within the same general MAO-B cavity but show candidate-specific combinations of polar, aromatic, alkyl, sulfur-associated and van der Waals contacts. Across the maps, recurrent cavity residues include Cys172, Ile198/Ile199, Tyr326, Phe343, Cys397, Tyr398 and Tyr435. This overlap is structurally meaningful because the set spans the entrance/substrate gate, the hydrophobic pocket, the covalent-FAD neighborhood and the aromatic cage.

The same residues define the deposited reference pocket. Coordinate analysis of the 6FW0 chain-A ligand E92 identified 14 protein residues with at least one heavy atom within 4.0 Å of the ligand, including Cys172 (3.43 Å), Ile198 (3.71 Å), Ile199 (3.30 Å), Gln206 (3.25 Å), Tyr326 (3.20 Å), Phe343 (3.72 Å), Tyr398 (3.59 Å) and Tyr435 (3.54 Å). The nearest E92–FAD heavy-atom separation was 3.90 Å, and the ligand occupies a box of approximately 7.1 × 8.2 × 7.2 Å. Cys397, which anchors FAD covalently, lies 8.78 Å from E92 and therefore serves as a functional reference outside the contact shell. The residue set highlighted by the candidate interaction maps thus coincides with the experimentally defined recognition environment of a nanomolar chromone inhibitor [20].

The docking search space was defined from the docked MAO-B–CID 143667968 complex (Section 4.3). The 27 ligand heavy atoms span 8.29 × 11.67 × 9.80 Å; expanded by 4 Å on each side, they define a search box of 16.29 × 19.67 × 17.80 Å centered at x = 51.29, y = 153.75, z = 26.68 Å. In this complex, 18 protein residues lie within 4.0 Å of the ligand (Gly58, Pro102, Trp119, Leu164, Leu167, Phe168, Leu171, Cys172, Ile198, Ile199, Gln206, Ile316, Tyr326, Leu328, Phe343, Tyr398, Gly434 and Tyr435), including all eight E92 contact residues listed above; by construction, every atom within 4.0 Å of the ligand lies inside the box, so the box encloses the experimentally defined recognition environment with a margin suited to the larger candidate ligands. Two close heavy-atom contacts (Ile199, 2.26 Å; Leu171, 2.65 Å) indicate a tight steric fit in the docked pose and were flagged for pose-validity analysis (Section 3).

The spatial distribution of these contacts was more informative than their total number. Interactions near Ile199/Tyr326 could alter the entrance-to-substrate cavity gate, whereas contacts around Cys397/Tyr398/Tyr435 could influence ligand orientation relative to FAD. Accordingly, residue-specific RMSF was examined alongside whole-protein RMSD.

### 2.6. Global MD Statistics

The 50–100 ns interval was analyzed to reduce the contribution of early relaxation (Table 6). Apo MAO-B and the CID 42876521 complex showed similar late RMSD means, while CID 143667968 showed broader dispersion and a continued upward trend, indicating residual conformational adjustment rather than improved convergence.

Five non-overlapping 10 ns blocks were used to assess temporal behavior within each trajectory. Block means remained within 0.427–0.434 nm for apo MAO-B and 0.423–0.440 nm for CID 42876521, whereas those of CID 143667968 rose from 0.393–0.397 nm in the first two blocks to 0.437 nm in the last. Block-averaging standard errors [45] increased with block length for both complexes (0.0024 to 0.0045 nm and 0.0057 to 0.0102 nm, respectively) without reaching a plateau, whereas that of apo MAO-B stayed at 0.0005–0.0013 nm (Table 6). The late series are therefore not stationary on the 50–100 ns timescale, and these standard errors are lower bounds and not converged precision estimates. Late RMSD means are accordingly reported descriptively: the 0.001 nm difference between CID 42876521 and apo lies within this uncertainty, and the lower mean of CID 143667968 is not interpreted as greater stability. Over the final 25 ns the RMSD slopes of the two complexes were similar (0.00158 and 0.00154 nm ns^−1^; apo −0.00022 nm ns^−1^), so the smaller drift of CID 42876521 over 50–100 ns depends on the chosen window and is not claimed as a stability advantage.

### 2.7. Rg, SASA, and Hydrogen Bonds

**Figure 6:**
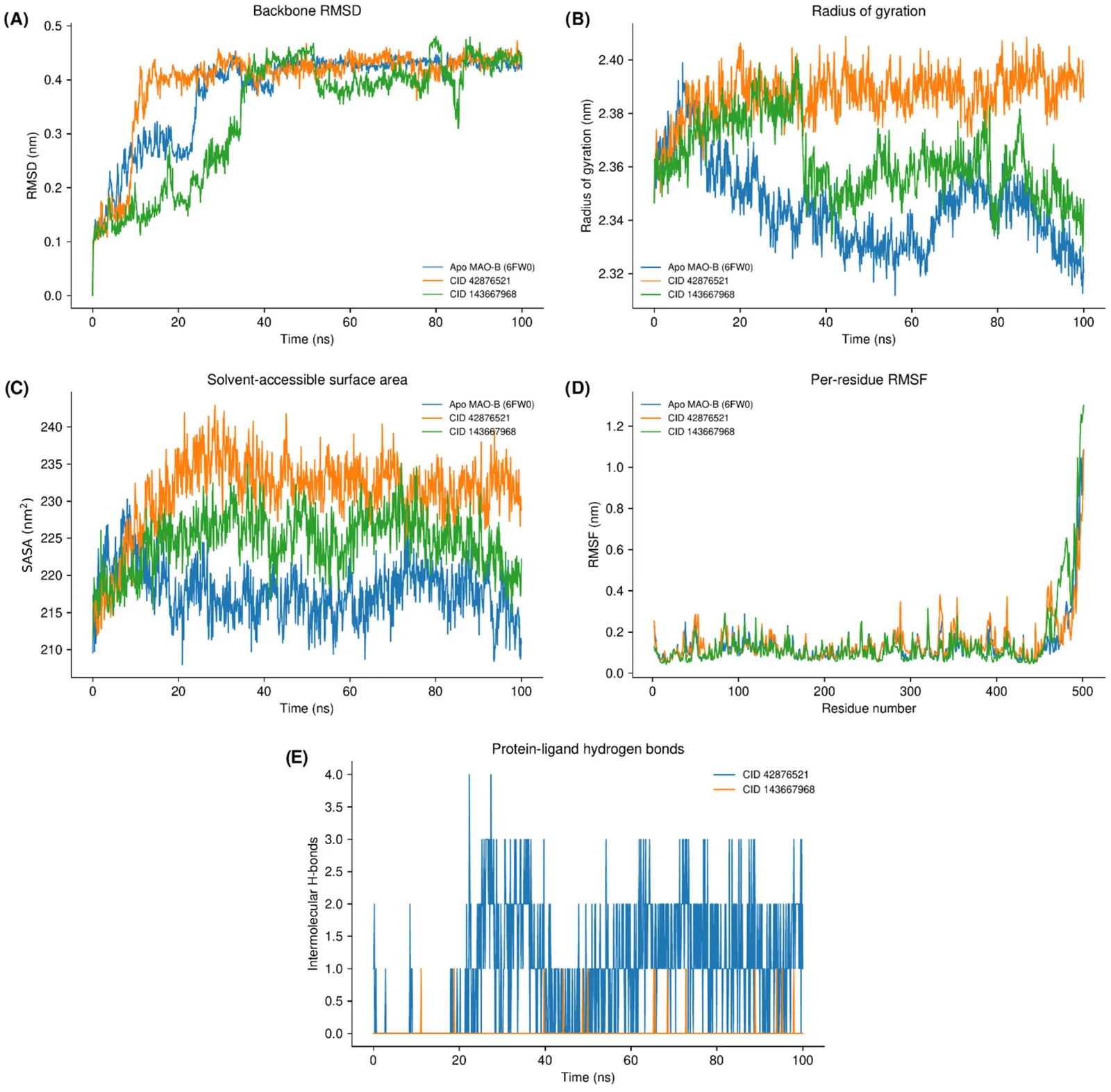
100 ns MD observables for apo MAO-B and the two MD-selected ligand complexes. (**A**) Backbone RMSD. (**B**) Radius of gyration (Rg). (**C**) Solvent-accessible surface area (SASA). (**D**) Per-residue RMSF. (**E**) Protein–ligand hydrogen-bond count.

Late Rg values were 2.3389 ± 0.0110 nm for apo MAO-B, 2.3895 ± 0.0067 nm for CID 42876521 and 2.3564 ± 0.0095 nm for CID 143667968, corresponding to increases over apo of 2.17% and 0.75%, respectively (Figure 6B). The block interval of CID 42876521 (2.3855–2.3936 nm) remained above that of apo (2.3275–2.3503 nm), indicating a consistently more expanded ensemble within this trajectory, whereas CID 143667968 showed a slightly negative late Rg slope (−0.000273 nm ns^−1^).

**Figure 7:**
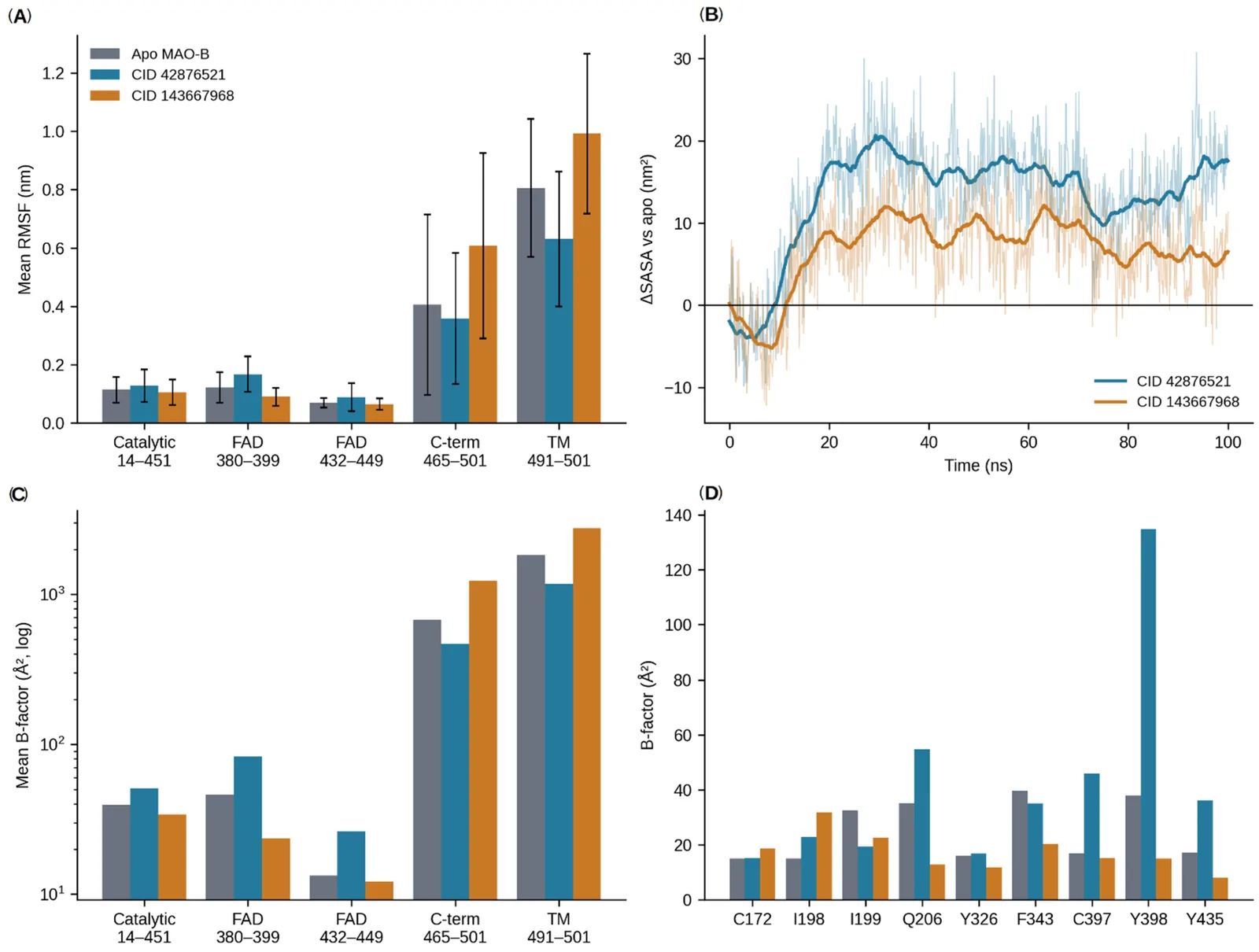
Domain- and residue-resolved dynamics of MAO-B. (**A**) Mean RMSF across residues within the Pfam catalytic domain, two FAD-facing PRINTS motifs, the C-terminal CATH region and the modeled transmembrane segment; error bars show SD across residues (residues are spatially correlated, so bars are descriptive). (**B**) ΔSASA (complex minus apo) over 100 ns; thin lines, raw data; thick lines, 5 ns running mean. (**C**) Regional mean B-factors derived from RMSF. (**D**) B-factors of selected gate, cavity and FAD-facing residues.

SASA changed in the same direction (Figure 6C). During 50–100 ns, mean SASA was 217.20 ± 3.29 nm^2^ for apo MAO-B, 231.96 ± 2.72 nm^2^ for CID 42876521 and 224.86 ± 3.42 nm^2^ for CID 143667968. Ligand binding therefore did not induce global compaction; both complexes occupied bounded ensembles that were modestly more expanded and solvent-exposed than apo, particularly CID 42876521. The difference from apo (ΔSASA) was +14.76 ± 0.93 nm^2^ (+6.8%) and +7.66 ± 1.44 nm^2^ (+3.5%), respectively (propagated SE), and the same ordering held in every window examined (0–100, 20–100, 50–100 and 75–100 ns). After an initial lag of about 10 ns, ΔSASA rose to a plateau within 20–30 ns for both complexes (Figure 7B). Only total SASA was exported, so ΔSASA cannot be partitioned between the protein and ligand-contact surface.

Hydrogen bond persistence differed markedly between the two complexes (Table 7; Figure 6E). For CID 42876521, at least one protein–ligand hydrogen bond was present in 61.1% of all frames, increasing to 82.4% over 50–100 ns (mean count 1.236 ± 0.803), with a maximum of four bonds over the full trajectory. CID 143667968, by contrast, averaged only 0.016 ± 0.1255 bonds over 50–100 ns (≥1 bond in 1.6% of frames; no frame with two or more). The contrast was preserved in all four windows examined (≥1-bond occupancy of 83.3% versus 1.6% over 75–100 ns), and block-averaging standard errors of the late mean bond count were 0.09–0.13 and 0.006–0.009, respectively (block lengths 5–12.5 ns). Hydrogen bonds were counted with the geometric criteria defined in Section 4.6. With one trajectory per complex, this contrast in exported hydrogen bond counts is the clearest difference between the two trajectories, but it is a descriptive observation and not a statistically resolved effect, and it does not establish the stronger affinity, inhibition or retention of the original binding pose.

### 2.8. Domain- and Residue-Resolved RMSF Suggest Two Distinct Local Dynamic Responses

Across the catalytic domain (residues 14–451), mean RMSF was higher for CID 42876521 (+12.1%) and lower for CID 143667968 (−8.2%) than for apo MAO-B (Table 8; Figure 7A). The difference became larger within the FAD-facing 380–399 motif (+37.3% and −26.3%, respectively) and was smaller in the 432–449 motif containing Tyr435 (+27.4% and −6.5%), consistent with increased local mobility for CID 42876521 and reduced mobility for CID 143667968 in the FAD-facing region of these trajectories. Per-residue RMSF profiles were strongly conserved across systems (Pearson r = 0.89 and 0.92 for CIDs 42876521 and 143667968 versus apo over all 500 residues; 0.79 and 0.77 over the catalytic domain), so the ligand-specific effects are superimposed on a shared fold-level fluctuation pattern.

The C-terminal region (residues 465–501) showed a different pattern. Relative to apo, mean RMSF was lower for CID 42876521 (−11.6%) and substantially higher for CID 143667968 (+49.9%), and the modeled transmembrane segment (491–501) reached the largest absolute values in all three systems (0.63–0.99 nm; Table 8). Because this segment is normally membrane anchored, the high terminal mobility reflects the missing lipid environment of the aqueous model rather than catalytic destabilization.

Per-residue RMSF was converted to isotropic B-factors, B = (8π^2^/3)·RMSF^2^ (RMSF in Å), to allow comparison with the crystallographic temperature factors of 6FW0 (Table 9; Figure 7C,D). The simulated B-factors reproduced the regional hierarchy of the RMSF analysis: within the FAD-facing 380–399 motif they increased by 80% for CID 42876521 and decreased by 49% for CID 143667968 relative to apo. At residue level, the Tyr398 B-factor increased from 37.9 Å^2^ (apo) to 134.9 Å^2^ with CID 42876521 and fell to 15.0 Å^2^ with CID 143667968, whereas Ile199 decreased in both complexes (32.7 Å^2^ to 19.2 and 22.6 Å^2^, respectively). The C-terminal region reached 466–1231 Å^2^, far above the crystallographic value (31 Å^2^), consistent with the missing membrane restraint. Over the catalytic domain the simulated profiles correlated moderately with the Cα B-factors of 6FW0 (Pearson r = 0.38–0.47) and were approximately 2–3 times higher in absolute terms; the B-factors are therefore used only as a relative measure among the three simulated systems.

Residue-level RMSF localized these differences to functionally relevant sites (Table 10; Figure 7D). In the CID 42876521 complex, Ile199 RMSF decreased by 23.3%, whereas Cys397, Tyr398 and Tyr435 increased by 64.9%, 88.7% and 45.4%, and Tyr326 was essentially unchanged (+2.1%). CID 143667968 instead reduced the fluctuations of Tyr326 (−14.0%), Phe343 (−28.5%), Tyr398 (−37.1%) and Tyr435 (−31.9%), and Ile199 decreased as well (−16.9%). The opposite Tyr398 and Tyr435 responses further distinguish the local dynamic effects of the two ligands.

### 2.9. DFT Identifies Electronic Complementarity Rather than a Direct Potency Ranking

The three ligands differed in their DFT-derived electronic descriptors (Table 11). CID 143667968 had the smallest HOMO–LUMO gap (ΔE = 2.421 eV) and CID 42876521 the largest (2.716 eV), with CID 1232631 intermediate (2.513 eV); hardness followed the same order, whereas softness followed the reverse order. All derived quantities were recomputed from the five-decimal orbital energies with a single convention (η = ΔE/2, S = 1/η, ω = μ^2^/2η) following Koopmans-type approximations [46,47,48,49]. Under this convention the electrophilicity indices are 0.5877, 0.4598 and 0.5269 a.u. for CIDs 143667968, 42876521 and 1232631 (half of the values obtained when the denominator is η rather than 2η), and the maximum electron-acceptance capacity ΔN_max_ = −μ/η is 5.14, 4.29 and 4.78, respectively.

**Figure 8:**
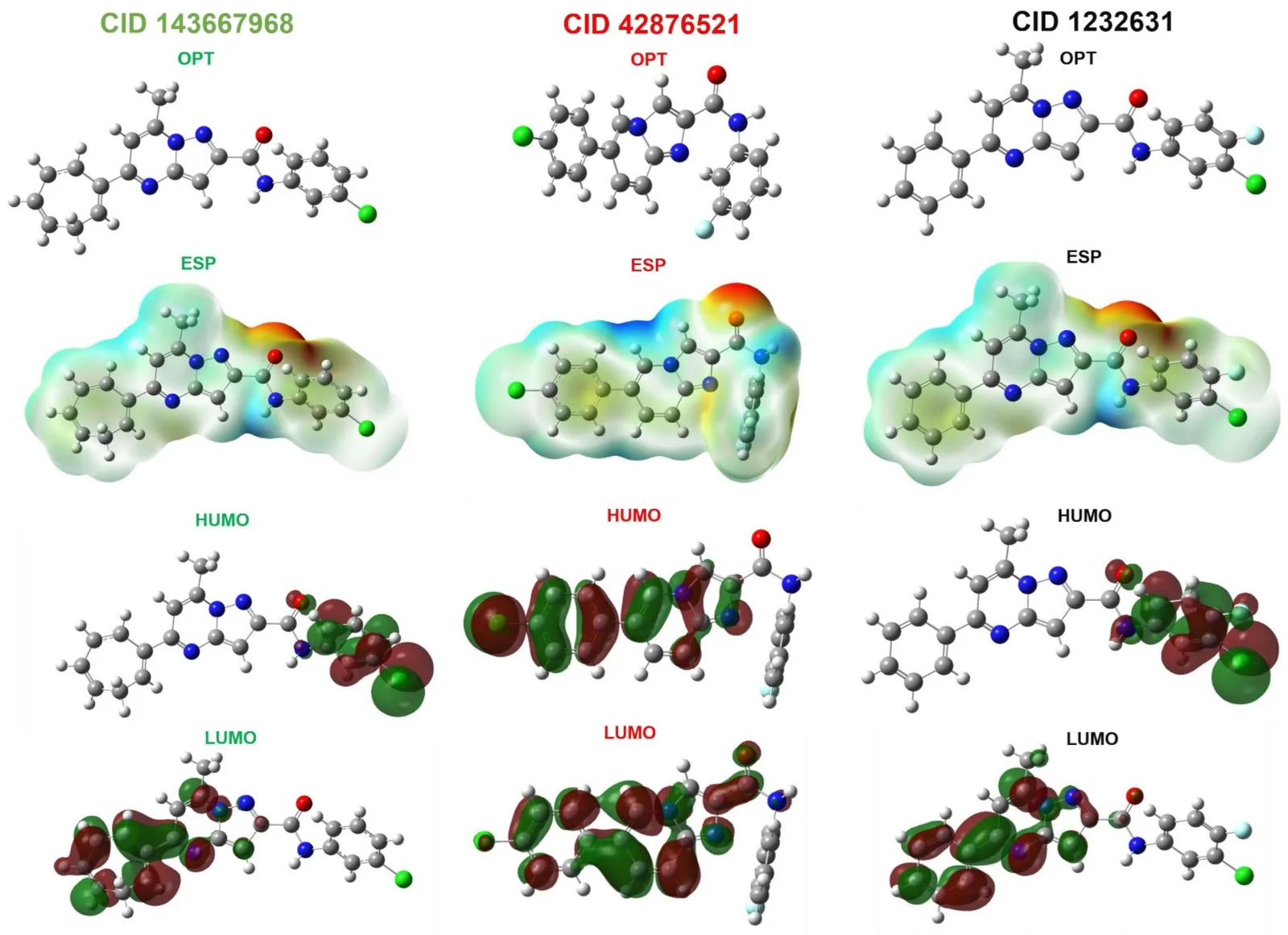
Comparative DFT visualization of optimized geometry (OPT), molecular electrostatic potential (ESP), HOMO, and LUMO for CIDs 143667968, 42876521, and 1232631. Orbital localization and ESP patterns describe intrinsic electronic organization and potential donor/acceptor regions. Atom colors follow the GaussView convention (C, gray; H, white; N, blue; O, red; F, pale cyan; Cl, green). In the ESP maps, red denotes more negative and blue more positive potential; in the HOMO/LUMO plots, the two lobe colors (green and red) denote opposite orbital phases.

**Figure 9:**
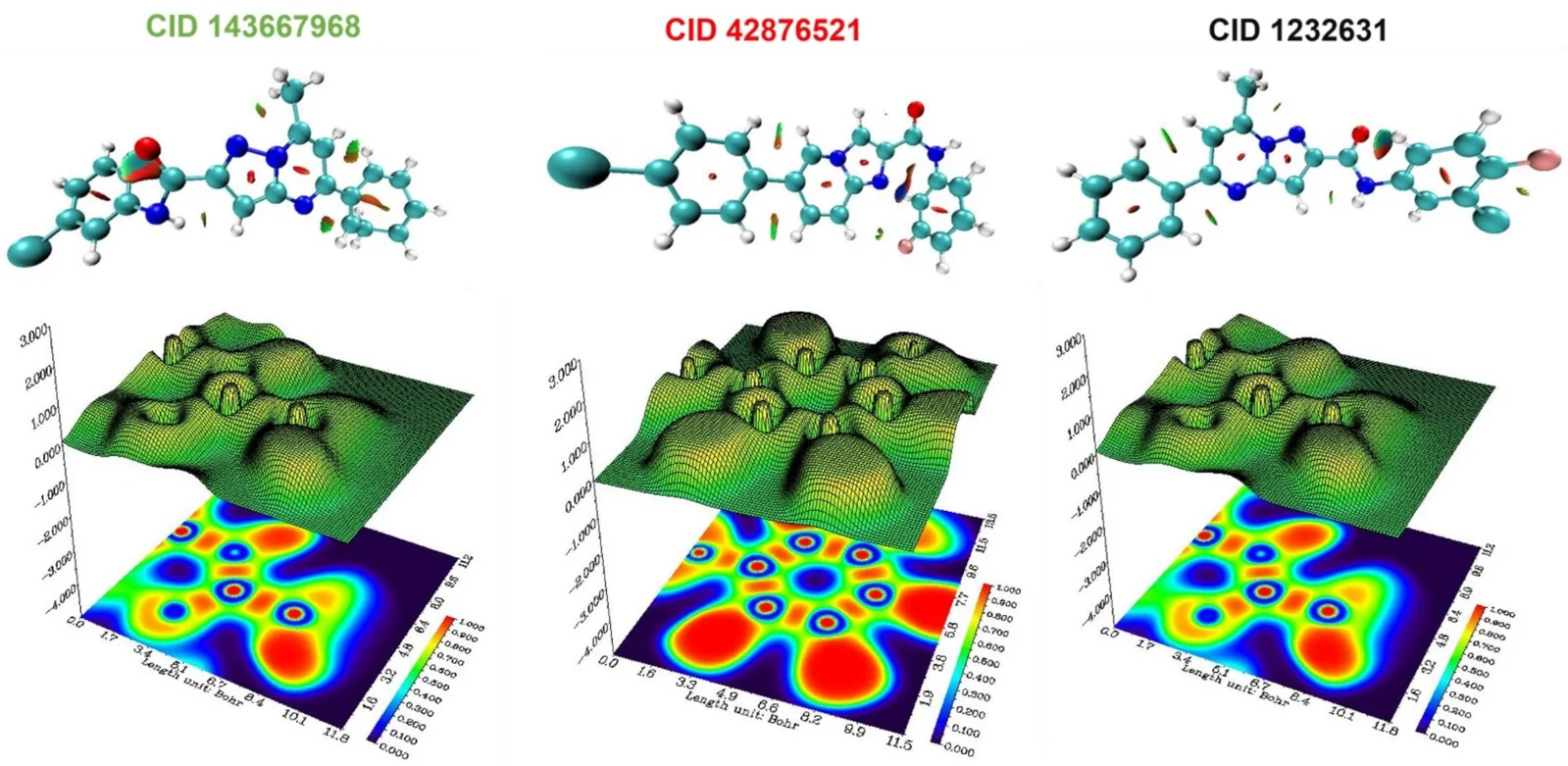
Noncovalent interaction and electron localization representations of the three prioritized ligands, shown from left to right as CIDs 143667968, 42876521 and 1232631. The upper molecular renderings show weak-interaction isosurfaces derived from the DFT analysis, and the lower panels show ELF surfaces/maps. In the weak-interaction isosurfaces, colors follow sign(λ2)ρ (blue, strongly attractive; green, weak van der Waals; red, repulsive/steric). The ELF color scale runs from 0.0 (blue) to 1.0 (red), as shown in the color bars.

**Figure 10:**
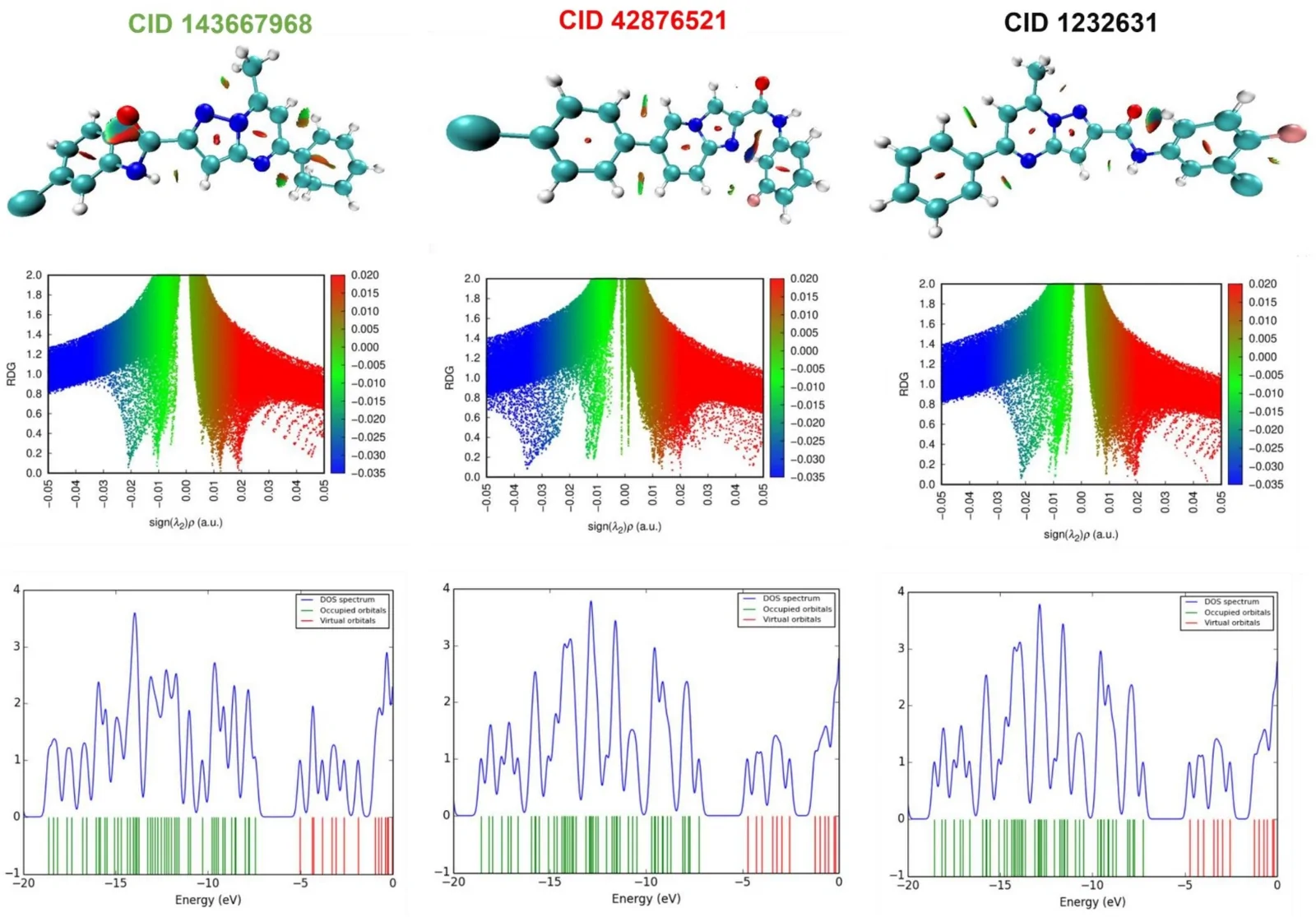
Reduced-density-gradient (RDG/NCI) and density-of-states (DOS) representations for CIDs 143667968, 42876521, and 1232631. RDG distributions describe attractive, dispersive, and sterically repulsive interaction regions, while DOS plots summarize occupied and virtual electronic states. In the RDG scatter plots, points are colored by sign(λ2)ρ (blue, attractive; green, weak van der Waals; red, repulsive), as shown in the color bars; in the DOS plots, the blue line is the DOS spectrum and green and red bars mark occupied and virtual orbitals, respectively.

Therefore, CID 143667968 is the most electronically soft and electrophilic compound of the set, whereas CID 42876521 is electronically the hardest. This electronic ordering differs from the MD hydrogen bond pattern, and is interpreted as complementary information on charge distribution and not as a potency ranking. The ESP maps (Figure 8) show pronounced electrostatic heterogeneity around carbonyl and heterocyclic heteroatoms, and the HOMO/LUMO, NCI/RDG, DOS and ELF visualizations (Figure 8, Figure 9 and Figure 10) provide the qualitative context for charge distribution and for the propensity for intramolecular and noncovalent interactions [50,51].

## 3. Discussion

This study links MAO-B target selection to pathway enrichment, structural annotation and ligand-dependent dynamics. MAO-B was not treated as a target solely because it is historically associated with PD; instead, the STRING/GO/KEGG data show that the selected MAOB neighborhood is quantitatively concentrated in the same biochemical space that motivates pharmacological inhibition. Of 11 network genes, 9 were assigned to amine metabolism, 7 to catecholamine metabolism and 6 to dopamine metabolism, while tyrosine metabolism produced the strongest KEGG signal (8 genes; FDR 1.42 × 10^−17^). This systems context is consistent with the clinical use of MAO-B inhibitors and with current PD treatment frameworks [1,2,5,14,52]. The network therefore provides a functional context for target selection, whereas the KEGG hsa:4129 annotation provides the curated link between MAOB and the Parkinson disease pathway hsa05012 [30,31]. Because the neighborhood was built around MAOB, the enrichment is read as the contextualization of an established target; the new information lies in the structure-resolved interpretation that follows.

Structural annotation defines the regions needed to interpret ligand-dependent dynamics. The Pfam amine-oxidase domain spans residues 14–451 and overlaps the FAD/NAD(P)-binding structural envelope, while the 380–399 PRINTS motif contains Cys397, which is covalently linked to FAD, immediately adjacent to Tyr398. Together with Tyr435, Tyr398 forms the aromatic environment that constrains substrate orientation near the flavin, whereas Ile199 and Tyr326 participate in the entrance/substrate cavity gate [2,53]. The E92 contact shell (14 residues within 4.0 Å, 3.90 Å from FAD) matches this residue set, which supports the choice of Cys172, Ile198/Ile199, Gln206, Tyr326, Phe343, Tyr398 and Tyr435 as the readout residues for RMSF. The pronounced RMSF rise near residues 465–501 occurs in the C-terminal CATH region and modeled transmembrane-anchor segment. Because the trajectories model chain A in aqueous solution rather than the native mitochondrial membrane, the high terminal RMSF is most plausibly attributable to the absence of membrane restraint rather than to a catalytic-site effect [15,23], in line with the documented coupling of MAO-B dynamics to its membrane environment [54].

CID 42876521 showed a late RMSD of 0.431 ± 0.015 nm, close to apo MAO-B (0.430 ± 0.008 nm), with five late 10 ns block means between 0.423 and 0.440 nm; this plateau is not interpreted as convergence, because the series are autocorrelated and the final-window RMSD slopes of the two complexes are similar. Its ≥1 protein–ligand hydrogen bond occupancy increased from 61.1% of all frames to 82.4% during 50–100 ns, and was 83.3% over 75–100 ns. Concurrently, Ile199 RMSF decreased by 23.3% relative to apo, while Cys397 and Tyr398 RMSF increased by 64.9% and 88.7%. One interpretation is that CID 42876521 maintains polar anchoring while reducing Ile199 mobility and permitting greater motion near the FAD-facing residues. The larger late Rg (+2.17% versus apo) and SASA (+6.79%) indicate a bounded but modestly more expanded and solvent-exposed ensemble rather than simple compaction. If this hydrogen bond pattern reflects the retention of the docked pose, which requires ligand RMSD and contact-occupancy analyses to confirm, polar anchoring would be a design-relevant feature, because hydrogen bonds with Cys172 and Tyr435 are part of the binding mode of the reference chromone [20], and directional polar contacts at the entrance cavity are also a hallmark of reversible inhibitors such as safinamide [55].

CID 143667968 showed a different dynamic pattern. Its late mean RMSD was numerically lower (0.412 ± 0.031 nm), but the coefficient of variation was 7.53% versus 3.41% for 42876521, and the late linear drift was 0.00104 nm ns^−1^ compared with 0.000317 nm ns^−1^. Its 10 ns block mean RMSD rose from 0.397 nm at 50–60 ns to 0.437 nm at 90–100 ns, consistent with ongoing conformational adjustment. Persistent hydrogen bonding was nearly absent (≥1 bond in 1.6% of late frames), while Tyr398 and Tyr435 RMSF decreased by 37.1% and 31.9% and Phe343 by 28.5%, consistent with local rigidification of the aromatic/hydrophobic cavity. This combination is compatible with a hypothesis of nonpolar recognition in the aromatic cage, a mode that resembles tight-binding chromone and coumarin inhibitors in the MAO-B substrate cavity [20,55], but it does not demonstrate hydrophobic packing: without ligand RMSD, ligand–pocket distances or contact occupancy, pose retention cannot be assessed. Ligand RMSD, pose clustering and contact-occupancy analyses will test this interpretation directly.

DFT and ADMET results show why the candidates are best judged on several scores simultaneously. CID 143667968 had the smallest HOMO–LUMO gap (0.08897 a.u.; 2.421 eV), the lowest hardness (0.044485 a.u.) and the highest electrophilicity, indicating greater electronic softness and charge responsiveness than 42876521, whose gap was 0.09981 a.u. (2.716 eV). Conversely, CID 42876521 had the strongest drug-likeness model score (0.73) and the highest exported hydrogen bond count. The nature of this complementarity is expected: classical MD does not model electronic redistribution, and frontier-orbital energies describe the isolated ligand, so a soft ligand with few hydrogen bonds and a hard ligand with many hydrogen bonds are mutually compatible outcomes.

Predicted Ames, carcinogenicity and hERG alerts apply to all three candidates, with an extra mouse carcinogenicity call for 42876521, and constitute the main hurdle on the path from computational lead to preclinical candidate. These endpoints are in silico classifications and are addressed in an established order: confirmation of the alerts with orthogonal predictors and an applicability-domain check, identification of the substructure responsible, and design of analogs that remove or mask the alerting motif while retaining the contact pattern defined by the MD analysis (the polar anchor for 42876521 and the aromatic-cage packing for 143667968). hERG liability is typically attenuated by reducing lipophilicity or basicity and by adding polar or ionizable handles that do not intrude on the MAO-B cavity [41,44]. The favorable TPSA and size of 42876521 leave ample property space for such modifications.

Established MAO-B inhibitors provide the pharmacological benchmark. Selegiline and rasagiline inhibit irreversibly, whereas safinamide exemplifies reversible noncovalent recognition [7,8,55]. The chromone series underlying 6FW0 was characterized by kinetics and crystallography, and the chlorophenyl compound corresponding to E92 shows low-nanomolar tight-binding inhibition [20]; potencies of this order are the target that the present candidates must approach in enzyme assays. The workflow is comparable in scope to recent computational MAO-B studies that combine docking, 100–200 ns MD, DFT and ADMET [27,28,29,56], and it adds the pathway layer and domain-resolved RMSF. Consistent with current practice for docking-derived hypotheses, the next experimental step is direct biochemical testing [57].

### Limitations and Future Directions

This study represents a preliminary computational investigation rather than experimental evidence of MAO-B inhibition or therapeutic potential. MAO-B/MAO-A inhibition, blood–brain barrier (BBB) transport, target engagement, in vivo exposure, and safety were not experimentally measured. BBB classifications were based on predictive tools and the available screening comparison, and their reliability across all shortlisted compounds remains uncertain.

PDB 6FW0. Additionally, the initial heavy-atom contacts involving Ile199 (2.26 Å) and Leu171 (2.65 Å) in the CID 143667968 pose require steric validation [58]. MD simulations were limited to single 100 ns aqueous simulations of chain A for two ligands, without independent replicas, membrane embedding, systematic ligand RMSD and contact-occupancy analyses, or free-energy calculations [59]. Consequently, the observed interactions remain preliminary structural observations rather than evidence of persistent binding or high affinity.

Future computational work should validate the docking protocol through native-ligand redocking, symmetry-aware heavy-atom RMSD, and steric-contact assessment. Selected compounds should be evaluated in plausible protonation and tautomeric states, with alternative scoring functions and reference inhibitors used to assess ranking robustness. At least three independently initialized MD trajectories per system should subsequently examine apo MAO-B, the two simulated compounds, and the untested backup CID 1232631, preferably in an outer-mitochondrial-membrane-mimetic environment. Analyses should assess ligand RMSD relative to the aligned binding pocket, residue-level contact occupancy, water-mediated interactions, conformational clustering, equilibration, and variability between replicas, focusing on Ile199, Tyr326, Tyr398, Tyr435, and neighboring FAD-facing residues. MM/GBSA, MM/PBSA, or relative free-energy calculations should be considered only after pose validation and adequate conformational sampling, as supportive rather than definitive affinity estimates [60].

Experimental validation should begin with concentration–response human MAO-B inhibition assays, MAO-A counter-screening, and IC_50_ determination, followed by the characterization of K_i_, inhibition mechanism, reversibility, and time dependence. Assay-interference and aggregation controls are also necessary. Predicted CNS exposure should be evaluated using validated permeability assays, such as PAMPA-BBB or endothelial cell models, alongside appropriate transporter/efflux, unbound fraction, and metabolic stability assessments. Because the shortlisted compounds carry predicted Ames, carcinogenicity, and hERG liabilities, these alerts require independent investigation rather than being treated as confirmed toxic effects. Bacterial reverse-mutation testing, cell viability assessment, and direct hERG electrophysiology should precede animal efficacy studies.

Subsequent hit-to-lead optimization should preserve experimentally validated MAO-B recognition features while reducing potential toxicophores and improving solubility, metabolic stability, and measured brain exposure. CID 42876521 is the initial testing priority because its existing trajectory showed frequent hydrogen bond interactions and its calculated ligand efficiency and drug-likeness descriptors were favorable. CID 143667968 provides a complementary candidate, although its close initial contacts require further structural assessment, while CID 1232631 remains an untested MD backup.

## 4. Materials and Methods

### 4.1. STRING, GO and KEGG Target Contextualization

Human MAOB was used as the seed for a first-shell STRING v12.0 functional association network (Homo sapiens; 10 first-shell interactors; default minimum interaction confidence 0.400 and all evidence channels active). The first-shell network comprised MAOB, ALDH2, AOC1, AOC2, AOC3, AOX1, COMT, DBH, DDC, LRTOMT and PRKN. STRING integrates multiple evidence channels; edges were therefore interpreted as functional associations and not automatically as direct physical binding events [30]. GO Biological Process and KEGG enrichment were evaluated using gene counts, background counts, enrichment signal and false-discovery rate (FDR) as reported by STRING, with the whole human proteome as background. The KEGG human MAOB entry (hsa:4129; KO K00274) was independently checked for curated pathway membership, including hsa00350 (tyrosine metabolism), hsa04728 (dopaminergic synapse) and hsa05012 (Parkinson disease) [31].

### 4.2. Retrieval of PDB Entry 6FW0, Domain Annotation, and Functional Residue Mapping

The human MAO-B structure 6FW0 was used as the structural reference. Its biological assembly is a two-chain homodimer with covalently bound FAD; the docking/MD workflow analyzed chain A. PDB numbering and InterPro positions had offset 0. Chain A contains modeled residues 2–501 and chain B residues 2–496. Domain annotation was performed with InterProScan 5 using default settings for Pfam, PRINTS, CATH-Gene3D, SUPERFAMILY, PANTHER and Phobius annotations [32,34,61]. The principal Pfam amine-oxidase catalytic domain PF01593 (IPR002937) spans residues 14–451. PRINTS PR00757 identified nine flavin-amine-oxidase motifs at 6–25, 92–114, 174–195, 197–219, 294–313, 380–399, 401–423, 432–449 and 462–484. CATH assigned FAD/NAD(P)-binding architecture 3.50.50.60 to residues 6–448 and C-terminal domain 6.10.250.130 to residues 465–517; Phobius predicted a transmembrane helix at 491–511, of which residues 491–501 are modeled in chain A. Functional interpretation focused on Cys397 (covalent FAD attachment), Tyr398/Tyr435 (aromatic cage), Ile199/Tyr326 (cavity gate) and additional cavity residues identified in the interaction maps [23,53]. For the crystallographic reference analysis, the deposited 6FW0 coordinates were retrieved from the RCSB Protein Data Bank; chain-A E92 heavy atoms were compared with chain-A protein heavy atoms and FAD (hydrogen atoms excluded), and a residue was classified as proximal when its minimum heavy atom separation from E92 was ≤4.0 Å.

### 4.3. Ligand Library and GNINA Screening

The pharmacophore-derived input collection was 2870 entries from ZINCPharmer [62], with PubChem identifiers used for compound tracking [63]. CIDs 143667968, 42876521 and 1232631 correspond to the molecular formulas C_21_H_17_ClN_4_O, C_20_H_13_ClFN_3_O and C_20_H_14_ClFN_4_O. Docking was performed against the 6FW0 active site with GNINA 1.0, combining Vina-family pose/energy estimates with CNN-based scoring [37,38,39]. Chain A was used after the removal of crystallographic waters and ligand E92 while preserving covalent FAD; the ligand-preparation reference pH was 7.4 and the ligands were energy-minimized. The screening box was constructed from the docked CID 143667968 complex by expanding the ligand–heavy-atom bounding box (8.29 × 11.67 × 9.80 Å) by 4 Å on each side, yielding 16.29 × 19.67 × 17.80 Å centered at (51.29, 153.75, 26.68) Å. The documented workflow used an exhaustiveness setting of 8, up to nine output poses per ligand and the default CNN ensemble.

Screening and shortlisting criteria. More negative Vina scores were interpreted as more favorable empirical docking scores, whereas higher CNN-affinity outputs were interpreted as more favorable model-estimated affinity. For the CNS-oriented shortlist, compounds were compared jointly on both predicted quantities and their categorical BBB-permeation. Among the six BBB-positive compounds explicitly recorded in the curated comparison embedded in the supplied screening export, CIDs 143667968, 42876521 and 1232631 had the three highest listed CNN-affinity estimates while retaining favorable Vina values.

### 4.4. Binding-Mode Analysis

Docked poses were visualized with PyMOL 2.5, BIOVIA Discovery Studio 2025 and PLIP [64,65,66], and PLIP was run with its default interaction-detection thresholds. Static interactions were classified as conventional or donor hydrogen bonds, π–π interactions, π–alkyl/alkyl interactions, sulfur-associated contacts and van der Waals contacts. The interaction maps were interpreted as pose-level hypotheses, and were cross-read with MD hydrogen bond persistence and residue-level RMSF rather than assumed to remain unchanged during the 100 ns trajectories.

### 4.5. ADME, Drug-likeness and Toxicity

SwissADME was used to predict physicochemical properties, gastrointestinal absorption, BBB permeability, skin permeation, solubility, Lipinski/Ghose/Veber/Egan/Muegge drug-likeness filters, bioavailability score, BOILED-Egg classes and radar descriptors [42,43]. PreADMET was used for preliminary toxicity classification. A predicted BBB-positive status was a prioritization consideration for this CNS-oriented screening.

The standard SwissADME drug-likeness filters (Lipinski, Ghose, Veber, Egan and Muegge) were interpreted using the software-defined criteria. Specifically, the Lipinski implementation [67] applies MW ≤ 500 g mol^−1^, HBD ≤ 5, HBA ≤ 10 and MLOGP < 4.15 [42]. The bioavailability radar favorable ranges are XLOGP3 approximately −0.7 to 5.0, MW 150–500 g mol^−1^, TPSA 20–130 Å^2^, ESOL logS ≥ −6, fraction Csp3 ≥ 0.25 and ≤ 9 rotatable bonds [42]. CNS-oriented MW < 450 g mol^−1^ and TPSA < 90 Å^2^ were descriptive comparisons [40,41]. The categorical BOILED-Egg GI/BBB classes were set by SwissADME [42,43].

Favorable Vina/CNN-affinity and predicted BBB classes supported inclusion in the present triage, but they did not override other liabilities. Discordant classifications—for example, BBB-positive but poor predicted solubility or positive mutagenicity/hERG alerts—were recorded explicitly, so retained candidates remained screening hypotheses requiring optimization and experimental testing. ESOL and SILICOS-IT outputs were separate rather than collapsed into an artificial solubility pass/fail threshold. PreADMET Ames and carcinogenicity outputs were treated as positive/negative predictions and hERG as the reported risk category, without inventing model probability cutoffs. Predicted mutagenicity, carcinogenicity and medium hERG alerts for all three are material constraints on development (Table 5).

Protonation and pH. The docking-preparation protocol recorded pH 7.4 as its nominal physiological reference (Section 4.3). The molecular representation was supplied to SwissADME and PreADMET, including input SMILES, charge and protonation state.

### 4.6. Molecular Dynamics Simulations

The MD workflow used GROMACS 2019 and the CHARMM36-family force field [68,69]. CHARMM-GUI was used for system preparation [70,71], TIP3P water for solvation [72] and K^+^/Cl^−^ ions for neutralization; ligand parameters were generated with CGenFF [73]. The protocol comprised 5000 steepest-descent minimization steps, 125 ps of NVT equilibration and 100 ns of NPT production at 310 K and 1 atm. The following values are the CHARMM-GUI GROMACS defaults: a 2 fs time step with LINCS constraints on hydrogen-bonded bonds [74], particle mesh Ewald electrostatics [75] with a 1.2 nm real-space cutoff, force-switched van der Waals interactions between 1.0 and 1.2 nm, the velocity-rescaling thermostat [76], the Parrinello–Rahman barostat [77] and a minimum protein–box-edge distance of 1.0 nm. The analyzed systems were apo 6FW0 chain A, MAO-B–CID 42876521 and MAO-B–CID 143667968. Trajectories were sampled at 1001 time points (0–100 ns) for RMSD, Rg, SASA and hydrogen bonds, and residue-wise RMSF was calculated for 500 modeled residues. RMSD was computed for backbone atoms after least-squares fitting to the starting structure, and protein–ligand hydrogen bonds were counted with the GROMACS geometric criteria (donor–acceptor distance ≤ 0.35 nm; hydrogen–donor–acceptor angle ≤ 30°).

Because only one trajectory existed per system, no trajectory frame was treated as an independent replicate and no between-system frame-level *p*-values were calculated. Global observables were summarized over 0–100 ns (1001 frames), 20–100 ns (801), 50–100 ns (501) and 75–100 ns (251 frames) using the mean, SD (sample convention), median, interquartile range, minimum, maximum, coefficient of variation (CV) and least-squares linear slope versus time. The 50–100 ns interval was also partitioned into five non-overlapping 10 ns temporal blocks, and block means were summarized by their range. Block-averaging standard errors were calculated for block lengths of 2.5, 5, 10 and 12.5 ns; a standard error that increases with block length was taken to indicate residual autocorrelation or drift, and is reported only as a lower bound [45]. Neither blocks nor windows were treated as independent replicates, and no claim of equilibrium or convergence is made. Pearson correlation coefficients were calculated between per-residue RMSF profiles. ΔSASA was calculated as the difference between the late-window mean SASA of each complex and that of the apo trajectory, with the uncertainty obtained by propagating the block-averaging standard errors of the two separate trajectories (SE_Δ_ = √(SE_complex_^2^ + SE_apo_^2^), using the largest standard error over block lengths of 5–12.5 ns), so that no frame pairing across trajectories was assumed. B-factors were derived as B = (8π^2^/3)·RMSF^2^ with RMSF in Å and compared with the Cα B-factors of chain A in the deposited 6FW0 file. Hydrogen bond persistence was reported as the mean count and the occupancy of ≥1, ≥2 and ≥3 bonds for each window. RMSF was mapped to the catalytic domain (14–451), the FAD-facing motifs 380–399 and 432–449, the C-terminal CATH region 465–501 and the modeled transmembrane segment 491–501. Residue-level RMSF values were analyzed for Cys172, Ile198, Ile199, Gln206, Tyr326, Phe343, Cys397, Tyr398 and Tyr435.

### 4.7. Density Functional Theory

DFT calculations were performed with Gaussian 09 (Revision C.01) at the B3LYP/6-311++G(d,p) level [78,79,80,81,82], in the gas phase for neutral closed-shell singlet molecules with default SCF and geometry-convergence criteria. Geometry optimization was followed by vibrational frequency analysis at the same level, which confirmed that the optimized structures are true minima (no imaginary frequencies). HOMO and LUMO energies, the frontier-orbital gap (ΔE), electron affinity, ionization potential, electronegativity, hardness, softness, electrophilicity and charge-transfer descriptors were calculated as ΔE = E_LUMO_ − E_HOMO_, I ≈ −E_HOMO_, A ≈ −E_LUMO_, χ = (I + A)/2, μ = −χ, η = (I − A)/2, S = 1/η, ω = μ^2^/(2η) and ΔN_max_ = −μ/η [46,48], with 1 a.u. = 27.2114 eV. Wavefunction post-processing for the ESP, reduced-density-gradient/noncovalent-interaction (RDG/NCI), density-of-states (DOS) and electron-localization-function (ELF) analyses was carried out with Multiwfn 3.8 (2023) [83], and the corresponding graphics were prepared with GaussView [84]. These graphics were interpreted as intrinsic electronic descriptors [48,50,51].

### 4.8. Software and Computational Resources

The software and database resources used in this study are listed in Table 12.

## 5. Conclusions

This preliminary study integrates MAOB-related pathway annotation, structure-resolved recognition-site analysis, GNINA screening, predicted BBB compatibility, ADMET, MD and DFT to nominate testable MAO-B inhibitor hypotheses beyond docking score alone. The current evidence places CIDs 143667968, 42876521 and 1232631 in a favorable model-based CNS-screening shortlist. Within the limited single-trajectory comparison, CID 42876521 showed persistent hydrogen-bond counts, supporting its priority for initial assays; CID 143667968 offers a complementary docking/electronic profile, whereas CID 1232631 remains a backup requiring experimental assessment. The predicted mutagenicity, carcinogenicity and hERG liabilities are substantial and require careful verification before lead advancement. The study fills a defined preliminary screening gap by integrating target/pathway relevance, functional structural sites, complementary docking estimates, CNS-oriented prediction and developability flags into a testable prioritization strategy. Auditable score/BBB provenance, native-ligand redocking, replicated membrane-aware MD, hMAO-A/hMAO-B enzyme assays, measured BBB transport and safety assays are future directions.

## Figures and Tables

**Table 1:** Selected GO biological process enrichment terms from the MAOB-centered STRING network.

GO Biological Process	Genes	% of 11	Signal	FDR
Amine metabolic process	9	81.8	6.45	1.59 × 10^−15^
Cellular biogenic amine metabolic process	8	72.7	5.69	2.11 × 10^−13^
Catecholamine metabolic process	7	63.6	5.97	6.49 × 10^−13^
Dopamine metabolic process	6	54.5	5.37	2.87 × 10^−11^
Dopamine catabolic process	4	36.4	3.90	6.52 × 10^−8^
Neurotransmitter metabolic process	4	36.4	2.95	2.10 × 10^−6^

**Table 2:** Selected KEGG enrichments supporting the pathway context of the MAOB-centered network.

KEGG Pathway	Genes	% of 11	Signal	FDR
Tyrosine metabolism	8	72.7	8.29	1.42 × 10^−17^
Tryptophan metabolism	5	45.5	4.15	4.25 × 10^−9^
Metabolic pathways	10	90.9	1.46	5.01 × 10^−9^
Phenylalanine metabolism	4	36.4	4.00	2.63 × 10^−8^
Histidine metabolism	3	27.3	2.50	1.75 × 10^−5^
Dopaminergic synapse	4	36.4	1.99	3.22 × 10^−5^

**Table 3:** Structural and functional annotations used for domain-resolved interpretation of 6FW0.

Annotation	Residues	Functional Interpretation
Pfam PF01593/IPR002937	14–451	Amine-oxidase catalytic domain
PRINTS PR00757 motif	380–399	Contains covalent-FAD Cys397 and Tyr398
PRINTS PR00757 motif	432–449	Contains aromatic-cage Tyr435
CATH 3.50.50.60	6–448	FAD/NAD(P)-binding structural envelope
CATH 6.10.250.130	465–517	C-terminal structural region
Phobius transmembrane helix	491–511	Mitochondrial membrane-anchor region; modeled to 501 in chain A

**Table 4:** GNINA scores of the three prioritized compounds.

PubChem CID	Formula	Heavy Atoms	CNN Affinity (pK)	Vina (kcal mol^−1^)	Difference from Top	Ligand Efficiency
143667968	C_21_H_17_ClN_4_O	27	7.39	−13.48	0.00	0.50
42876521	C_20_H_13_ClFN_3_O	26	7.08	−13.26	+0.22	0.51
1232631	C_20_H_14_ClFN_4_O	27	7.07	−13.15	+0.33	0.49

**Table 5:** Selected ADMET, drug-likeness, and toxicity predictions for the three prioritized compounds.

Property	CID 143667968	CID 42876521	CID 1232631
MW (g mol^−1^)	376.84	365.79	380.80
TPSA (Å^2^)	59.29	46.40	59.29
ESOL logS	−5.15	−5.77	−5.21
SILICOS-IT logS	−5.94	−7.96	−7.97
Predicted GI absorption/BBB permeation	High/Yes	High/Yes	High/Yes
Bioavailability score	0.55	0.55	0.55
Drug-likeness model score	−0.06	0.73	0.02
Ames/hERG	Mutagen/medium risk	Mutagen/medium risk	Mutagen/medium risk
Carcino mouse/rat	Negative/positive	Positive/positive	Negative/positive

**Table 6:** Late-trajectory backbone RMSD statistics (one trajectory per system; descriptive). Block-mean range refers to five consecutive 10 ns blocks within 50–100 ns; block standard errors are given for block lengths of 2.5, 5, 10 and 12.5 ns and increase with block length for both complexes, so they are lower bounds. The last column gives the SD of the residuals after linear detrending.

System	RMSD 50–100 ns Mean ± SD (nm)	CV (%)	Slope (nm ns^−1^)	10 ns Block-Mean Range (nm)	Block SE (nm) at 2.5/5/10/12.5 ns	SD After Detrending (nm)
Apo 6FW0	0.4297 ± 0.0078	1.82	0.000011	0.4266–0.4343	0.0009/0.0011/0.0013/0.0005	0.0078
CID 42876521	0.4308 ± 0.0147	3.41	0.000317	0.4226–0.4401	0.0024/0.0032/0.0035/0.0045	0.0140
CID 143667968	0.4116 ± 0.0310	7.53	0.001041	0.3928–0.4374	0.0057/0.0061/0.0081/0.0102	0.0271

**Table 7:** Protein–ligand hydrogen bond counts and temporal occupancy.

System/Interval	Mean H-Bonds	≥1 (%)	≥2 (%)	≥3 (%)	Maximum
42876521, 0–100 ns	0.952	61.1	28.1	5.8	4
42876521, 20–100 ns	1.171	74.8	34.8	7.2	4
42876521, 50–100 ns	1.236	82.4	35.5	5.6	3
42876521, 75–100 ns	1.227	83.3	33.9	5.6	3
143667968, 0–100 ns	0.014	1.4	0	0	1
143667968, 20–100 ns	0.015	1.5	0	0	1
143667968, 50–100 ns	0.016	1.6	0	0	1
143667968, 75–100 ns	0.016	1.6	0	0	1

**Table 8:** Descriptive domain-resolved RMSF. SD is across residues within each correlated region.

Region	Apo RMSF (nm)	42876521	Change vs. Apo	143667968	Change vs. Apo
Catalytic domain 14–451	0.1142 ± 0.0445	0.1280 ± 0.0553	+12.1%	0.1049 ± 0.0437	−8.2%
FAD motif 380–399	0.1218 ± 0.0525	0.1671 ± 0.0608	+37.3%	0.0897 ± 0.0311	−26.3%
FAD motif 432–449	0.0693 ± 0.0165	0.0883 ± 0.0481	+27.4%	0.0648 ± 0.0201	−6.5%
C-terminal 465–501	0.4053 ± 0.3090	0.3582 ± 0.2239	−11.6%	0.6077 ± 0.3180	+49.9%
Modeled TM 491–501	0.8051 ± 0.2365	0.6303 ± 0.2313	−21.7%	0.9920 ± 0.2744	+23.2%

**Table 9:** Mean B-factors (Å^2^) derived from simulated RMSF compared with the Cα B-factors of deposited 6FW0 chain A.

Region	Apo	CID 42876521	CID 143667968	6FW0 Crystal
Catalytic domain 14–451	39.5	51.1	34.0	15.7
FAD motif 380–399	45.9	82.7	23.6	14.3
FAD motif 432–449	13.3	26.3	12.1	13.5
C-terminal 465–501	676.7	466.1	1230.8	31.3
Modeled TM 491–501	1839.6	1173.8	2770.2	51.2

**Table 10:** RMSF (nm) of selected gate, cavity, and FAD-facing residues.

Residue	Apo	CID 42876521	% vs. Apo	CID 143667968	% vs. Apo
Cys172	0.0755	0.0758	+0.4	0.0841	+11.4
Ile198	0.0754	0.0930	+23.3	0.1100	+45.9
Ile199	0.1114	0.0855	−23.3	0.0926	−16.9
Gln206	0.1156	0.1443	+24.8	0.0698	−39.6
Tyr326	0.0780	0.0796	+2.1	0.0671	−14.0
Phe343	0.1227	0.1154	−6.0	0.0877	−28.5
Cys397	0.0801	0.1321	+64.9	0.0760	−5.1
Tyr398	0.1200	0.2264	+88.7	0.0755	−37.1
Tyr435	0.0806	0.1172	+45.4	0.0549	−31.9

**Table 11:** Frontier-orbital energies and global reactivity descriptors computed from five-decimal orbital energies (a.u.; ΔE also in eV).

CID	HOMO	LUMO	ΔE	ΔE (eV)	Hardness η	Softness S	ω	χ	ΔN_max_
143667968	−0.27315	−0.18418	0.08897	2.421	0.044485	22.48	0.5877	0.2287	5.14
42876521	−0.26413	−0.16432	0.09981	2.716	0.049905	20.04	0.4598	0.2142	4.29
1232631	−0.26676	−0.17441	0.09235	2.513	0.046175	21.66	0.5269	0.2206	4.78

**Table 12:** Software and database resources in this study.

Software/Resource	Version	Supplier/Developer
BIOVIA Discovery Studio	2025	Dassault Systèmes BIOVIA, San Diego, CA, USA
PyMOL Molecular Graphics System	2.5	Schrödinger, LLC, New York, NY, USA
Gaussian	Gaussian 09, Revision C.01	Gaussian, Inc., Wallingford, CT, USA
Multiwfn	3.8 (2023)	Freely available at http://sobereva.com/multiwfn/, accessed on 21 October 2026
GNINA	1.0	Koes Group, University of Pittsburgh, Pittsburgh, PA, USA
GROMACS	2019	GROMACS development team (open source)
InterProScan	5	EMBL-EBI, Hinxton, UK
STRING	12.0	STRING Consortium (SIB, CPR, EMBL), https://string-db.org, accessed on 21 October 2026
SwissADME	Web service	SIB Swiss Institute of Bioinformatics/University of Lausanne
PreADMET	Web service	BMDRC, https://preadmet.webservice.bmdrc.org, accessed on 21 October 2026
ZINCPharmer	Web service	ZINCPharmer pharmacophore search
PubChem	Web service	NCBI, https://pubchem.ncbi.nlm.nih.gov, accessed on 21 October 2026
RCSB Protein Data Bank (PDB 6FW0)	-	RCSB PDB, https://www.rcsb.org, accessed on 21 October 2026
KEGG	Online database	Kanehisa Laboratories, https://www.kegg.jp, accessed on 21 October 2026
PLIP	Web service	Protein–Ligand Interaction Profiler
CHARMM-GUI	Web service	CHARMM-GUI
CGenFF	4.0	CHARMM General Force Field
GaussView	6	Gaussian, Inc.
Pfam/PRINTS/CATH-Gene3D/Phobius	Web service	Sequence and structure annotation databases

## Data Availability

Original outputs are available from the corresponding authors on reasonable request.

## Abbreviations

The following abbreviations are used in this manuscript:

Abbreviation	Full term
ADMET	Absorption, Distribution, Metabolism, Excretion, and Toxicity
BBB	Blood–Brain Barrier
CATH	Class, Architecture, Topology, Homologous superfamily
CNN	Convolutional Neural Network
DCCM	Dynamic Cross-Correlation Matrix
DFT	Density Functional Theory
DOS	Density of States
ELF	Electron Localization Function
ESP	Electrostatic Potential
FAD	Flavin Adenine Dinucleotide
FDR	False Discovery Rate
GO	Gene Ontology
GNINA	Molecular docking program with CNN-based scoring (the name is not an acronym)
hERG	Human Ether-à-go-go-Related Gene
HOMO	Highest Occupied Molecular Orbital
KEGG	Kyoto Encyclopedia of Genes and Genomes
LUMO	Lowest Unoccupied Molecular Orbital
MAO-A	Monoamine Oxidase A
MAO-B	Monoamine Oxidase B
MAOB	Monoamine Oxidase B gene
MD	Molecular Dynamics
MM/GBSA	Molecular Mechanics/Generalized Born Surface Area
MM/PBSA	Molecular Mechanics/Poisson–Boltzmann Surface Area
NCI	Non-Covalent Interaction
NVT	Constant Number of particles, Volume, and Temperature ensemble
NPT	Constant Number of particles, Pressure, and Temperature ensemble
PD	Parkinson’s Disease
PDB	Protein Data Bank
Pfam	Protein Families Database
PRINTS	Protein Fingerprints Database
RDG	Reduced Density Gradient
Rg	Radius of Gyration
RMSD	Root Mean Square Deviation
RMSF	Root Mean Square Fluctuation
SASA	Solvent Accessible Surface Area
STRING	Search Tool for the Retrieval of Interacting Genes/Proteins
TPSA	Topological Polar Surface Area
TM	Transmembrane
Vina	AutoDock Vina Scoring Function (Vina v1.2.7)
ΔE	Frontier Molecular Orbital Energy Gap
